# A perspective: some relationships between the biochemistry of photosynthesis and the gas exchange of leaves (*Planta* 153, 376–387)

**DOI:** 10.1007/s00425-025-04761-7

**Published:** 2025-07-09

**Authors:** Susanne von Caemmerer, Graham D. Farquhar

**Affiliations:** Division of Plant Sciences, Research School of Biology, The Australian National University, Acton, ACT 2600 Australia

**Keywords:** CO_2_ assimilation, Photosynthesis, Gas exchange, Rubisco, Chloropalst electron transport

## Abstract

The Planta paper “Some relationships between the biochemistry of photosynthesis and the gas exchange of leaves” explored the relationship between gas exchange measurements of CO_2_ assimilation rate and the in vitro activity of Rubisco and chloroplast electron transport capacity. It showed that *A*/*C*_i_ curves, the response of CO_2_ assimilation rate, *A*, to intercellular CO_2_ partial pressure, *C*_*i*_, were an ideal tool to capture the underlying photosynthetic biochemistry and could be used to quantify maximum Rubisco activity and electron transport capacity in vivo. We also derived the equations required to calculate *C*_*i*_ using a ternary diffusion model which are now used world-wide in portable gas exchange systems. Below we highlight the major findings reported in this paper and how they continue to influence current research.

## Introduction

**Fig. 1:**
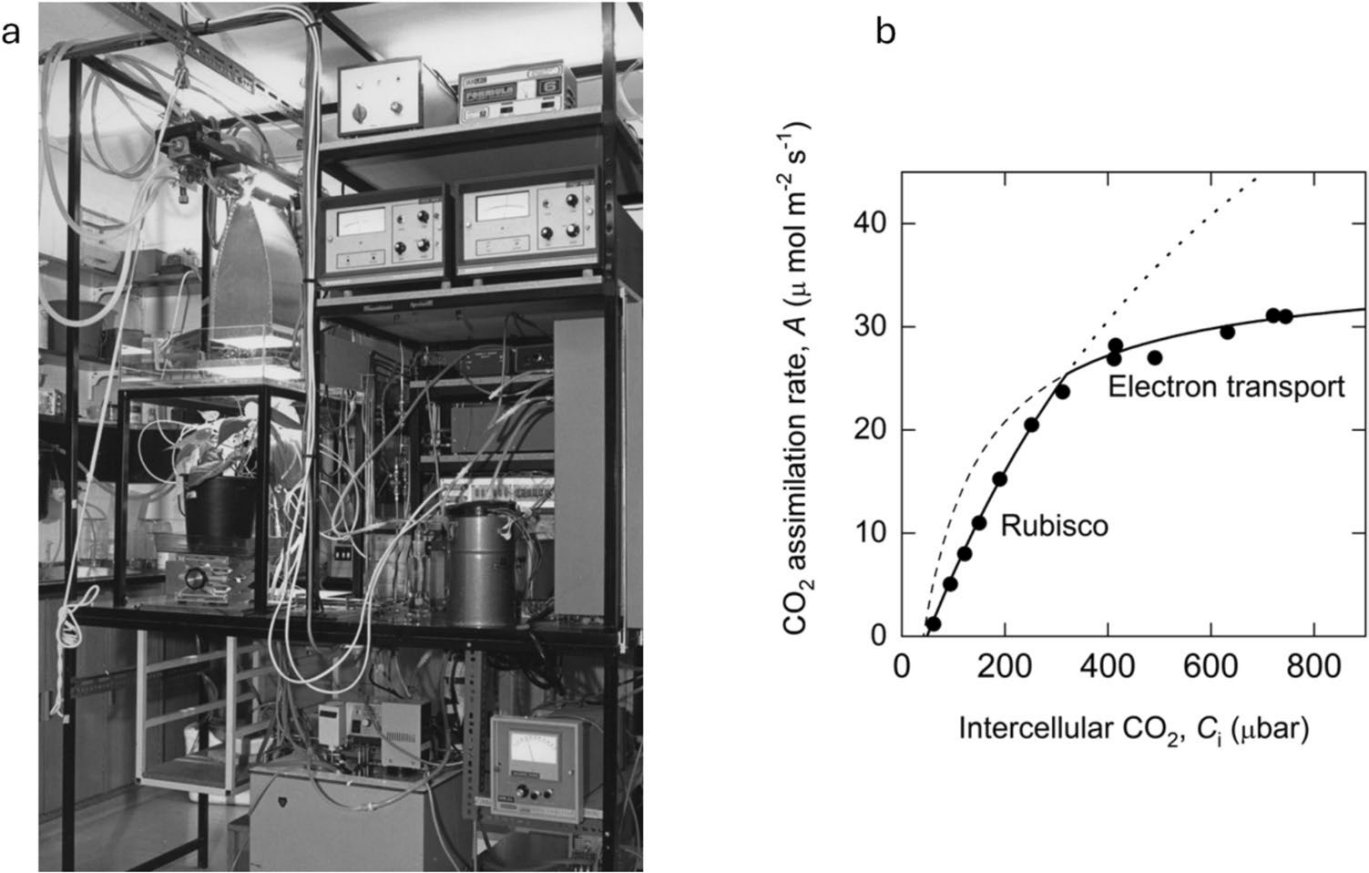
(**a**) Gas exchange system built by Suan Chin Wong at the Research School of Biological Sciences (Wong et al. 1978) used in the experiment by Susanne von Caemmerer for the data collected for (von Caemmerer and Farquhar 1981). An illuminated *Phaseolus vulgaris* plant can be seen illuminated with a xenon arc lamp with heat modified by a water bath. (**b**) Modelled response of CO_2_ assimilation rate as a function of intercellular [CO_2_].The dotted line and its extension represent the Rubisco limited (RuBP saturated) rate of CO_2_ assimilation and the dashed line and its extension the electron transport limited rate of CO_2_ assimilation. Data were from von Caemmerer et al. (1994) on a tobacco leaf

Modern analytical gas exchange is often attributed to the work of Pieter Gaastra (Gaastra 1959) who made the first measurements of CO_2_ assimilation and water vapour exchange rates on individual leaves. It started an enthusiasm for gas exchange measurements and a great body of work was accumulated looking at CO_2_ uptake and water loss of leaves under different environmental conditions (e.g. Björkman et al. 1972). A pivotal event in our understanding of the biochemistry of CO_2_ assimilation was the discovery by George Bowes and Bill Ogren that O_2_ was a competitive inhibitor of CO_2_ fixation of Rubisco and an alternative substrate leading to a side reaction that fuelled photorespiration (Bowes and Ogren 1972). The C_3_ photosynthetic model by Farquhar, von Caemmerer, and Berry captures this and links equations describing Rubisco kinetics with the stoichiometry of the carbon reduction cycle and the photorespiratory carbon oxidation cycle. In particular, the stoichiometry of ATP and NADPH requirements were linked to the light reactions (Farquhar et al. 1980). A key feature of the model was the suggestion that CO_2_ assimilation rate, *A*, could be described as either limited by the RuP_2_ saturated rate of Rubisco or the RuP_2_ regeneration/electron transport limited rate. This was based on the realisation that Rubisco was present in high concentrations in the chloroplast and that, therefore, in vivo Rubisco kinetics with respect to free and enzyme-bound RuP_2_ could be described by equations for a tight binding inhibitor or substrate (Farquhar 1979; Farquhar et al. 1980). A review of the model is given by Farquhar et al. (2001). Susanne von Caemmerer was a PhD student in Graham Farquhar’s laboratory at the time and her brief was to test this C_3_ photosynthetic model experimentally. We were interested in the extent to which observations made using biochemical techniques matched those made making measurements of leaf gas exchange. This involved two types of experiments. The first was concerned with the response of CO_2_ assimilation rate, *A*, to intercellular CO_2_ partial pressure, *C*_*i*_, as affected in the short-term by variation in irradiance, O_2_ partial pressure, and temperature. These curves are now called *A*/*C*_i_ curves. In the second set of experiments, photosynthesis of leaves was changed in the longer term through alterations in nitrogen nutrition, light environment, and changes in leaf age. Measurements of gas exchange were linked with biochemical measurements of in vitro Rubisco activity and electron transport rate made on isolated thylakoids on the same leaf. The gas exchange system used in these experiments is shown in Fig. 1a. It was built at the Research School of Biological Sciences by Suan Chin Wong. A key feature was that it had leaf clamp-on chambers which allowed a small section of a leaf to be measured (Wong 1979). Measuring small sections of the leaf required more precise instruments but makes it likely that cells measured behave similarly.

## Key findings of the paper

**Fig. 2:**
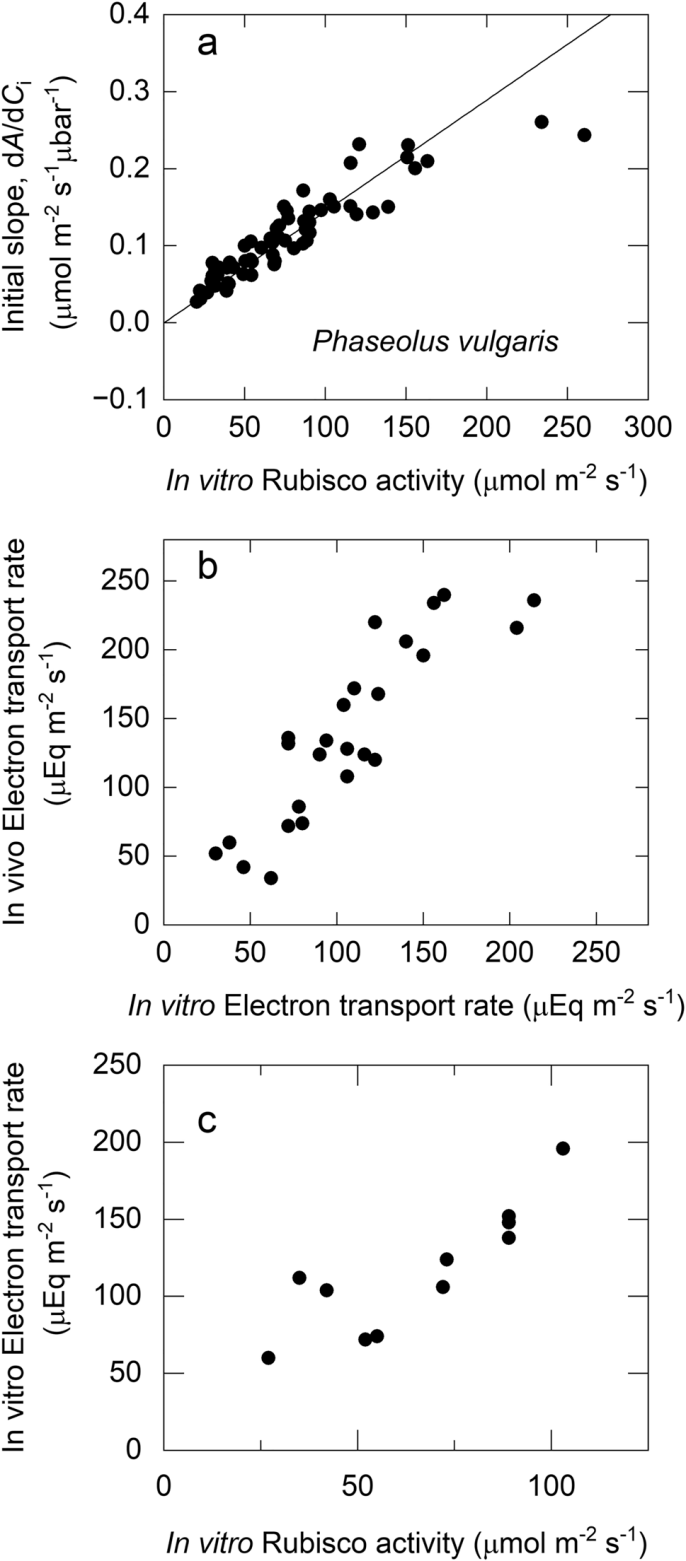
(**a**) The initial slope *(dA/dC*_*i*_) of the response of CO_2_ assimilation rate (*A*) to intercellular [CO_2_] (*C*_i_) versus in vitro measurements of Rubisco activity in *Phaseolus vulgaris*. Measurements were made at 28 °C and the source of variation in Rubisco activity were leaf age, nitrogen nutrition and growth irradiance. Data are taken from von Caemmerer and Farquhar (1981) and redrawn by von Caemmerer (2000). The line shows the relationship predicted by the C_3_ photosynthesis model by Farquhar et al. (1980) with updated Rubisco kinetic constants presented in von Caemmerer (2000). (**b**) Potential rate of electron transport, calculated from the response of CO_2_ assimilation rate to *C*_i_ vs in vitro electron transport rate (measured with methyl viologen as electron acceptor) in *P. vulgaris* grown in different nitrogen nutrition and light environments. The figure is redrawn from fig. 6 in von Caemmerer and Farquhar (1981). **c** In vitro measurements of electron transport rate vs in vitro Rubisco activity from leaves of *P. vulgaris* grown at different nitrogen nutrient. Redrawn from fig. 7 in von Caemmerer and Farquhar (1981)

1. The major hypothesis of the C_3_ photosynthesis model that CO_2_ assimilation rate was determined by the RuP_2_ saturated rate of Rubisco at low *C*_*i*_ and by the capacity of the chloroplast electron transport at high C_i._ was confirmed by short-term measurements of *A*/*C*_i_ curves at different irradiances, temperatures, and O_2_ partial pressures (Fig. 1b).
2. A strong quantitative relationship between the initial slope of the *A*/*Ci* curve with in vitro measurement of maximal Rubisco activity, *V*_*cmax*_, was shown in leaves of different photosynthetic capacities (Fig. 2a). It banished the myth that the amount of Rubisco in a leaf was in excess by showing that *V*_*cmax*_, had to be approximately four times the CO_2_ assimilation rate at ambient CO_2_.
3. In vitro rate of electron transport from H_2_O to methyl viologen measured on isolated thylakoids was quantitatively correlated with potential electron transport, *J*, calculated from CO_2_ assimilation rate at high *C*_i_ and high irradiance (Fig. 2b)
4. A close correlation between maximal Rubisco activity, *V*_*cmax*_, and electron transport rate, *J*, or *J*_*max*_* (*occurring at saturating irradiance) is shown in Fig. 2c. Interestingly, this close correlation is almost universal and difficult to perturb (von Caemmerer and Farquhar 1984; Wullschleger 1993).
5. A full set of equations required to calculate gas exchange parameters such as *C*_*i*_ is presented. The equations were derived using a ternary diffusion model accounting for the large fluxes of water vapour escaping the stomatal pore relative to the smaller CO_2_ fluxes entering the pore. These equations are now universally used and embedded in many portable gas exchange systems.

## Contribution to current research

### The application of A/Ci curves

The paper highlighted the usefulness of *A*/*C*_i_ curves for the assessment of photosynthetic biochemistry in C_3_ species. Examining *A* as a function of *C*_*i*_ rather than ambient [CO_2_] made it possible to separate stomatal and biochemical influences on *A*. The experiments in von Caemmerer and Farquhar (1981) were made with *Phaseolus vulgaris* where it was possible to extract functional Rubisco and thylakoids which allowed the establishment of the link between biochemistry and gas exchange. The two limitations to CO_2_ assimilation, *V*_*c*max_ and *J*_max_, are routinely reported in the literature using fitting routines developed for the analysis of *A*/*C*_i_ curves (Sharkey et al. 2007; Duursma 2015; Lochocki et al. 2025). *J*_max_ often refers incorrectly to electron transport rate measured at a fixed irradiance rather than a true maximal electron transport which is more difficult to measure. It would be useful if the irradiance at which these measurements were made be reported along with the fitted value by referring to this as *J*_1200_, for example if the measurements were made at an irradiance of 1200 µmol m^−2^ s^−1^ (for discussion, see Buckley and Diaz-Espejo 2015). It is, of course, also important to realise that, in some cases, *J* can also reflect a Calvin cycle limitation (Harrison et al. 2001). A third limitation at high *C*_i_ is called a triose phosphate limitation, it has been added later to explain the sometime-less–than-expected increase in *A* from a *J* limitation at high *C*_i_ (Harley and Sharkey 1991; Sharkey et al. 2007). Portable gas exchange systems have encouraged field experiments in crop and ecophysiology (Kattge et al. 2009). These measurements of *A*/*C*_i_ curves have made an important contribution to estimates of terrestrial photosynthesis in earth system models (Rogers et al. 2017).

### The importance of water vapour saturation in the leaf for the calculation of C_i_

The derivation of the gas exchange equations presented in Appendix 2 made the important assumption that the intercellular airspaces of leaves are saturated with water vapour. Saturated vapour pressure can be calculated from leaf temperature and this assumption is universally employed in all commercial portable gas exchange systems to calculate stomatal conductance and *C*_*i*_*.* However, recent research has shown that this may not always be the case, especially under conditions of a large water vapour difference between leaf and air (Wong et al. 2022; Cernusak et al. 2024). Incorrectly assuming water vapour saturation within the leaf results in underestimation of stomatal conductance and *C*_*i*_. Other extensions to the gas exchange equations have been the inclusion of the effect of cuticular conductance (Márquez et al. 2021). Another key assumption made in the calculation of *C*_*i*_ is that stomata behave uniformly across the leaf. Chlorophyll fluorescence imaging of leaves, however, have highlighted patchy behaviour (Mott and Buckley 2000). Although it is very useful to calculate *C*_*i*_ to get closer to leaf biochemistry, some caution is needed when gas exchange is measured under extreme conditions.

### Conservation of *J*_max_/*V*_*c*max_

Von Caemmerer and Farquhar noted that in all different growth experiments, they examined the ratio of *V*_*c*max_ and *J*_max_ was conserved in *Phaseolus vulgaris* (Fig. 2c; von Caemmerer and Farquhar 1981, 1984). This has since been confirmed by many other studies (Wullschleger 1993; Poorter and Evans 1998; Ainsworth and Long 2005; Yamori et al. 2010). This coordination between Rubisco and electron transport capacity led to the link between the in vivo estimates of Rubisco activity (*V*_*c*max_) and leaf nitrogen content to be used for parameterisation of global-scale terrestrial biosphere models (Wullschleger 1993; Kattge et al. 2009; Rogers et al. 2017). Only transgenic manipulations of the photosynthetic machineries in tobacco discussed below have achieved a gross imbalance (Hudson et al. 1992; Harrison et al. 2001).

### Transgenic manipulation of photosynthesis

In the 1990’s, transgenic manipulation became a tool to investigate limiting steps of photosynthesis, and many studies used tobacco as a model plant as it was easy to transform (Hudson et al. 1992; Price et al. 1998; Raines et al. 2000). Transgenic tobacco with antisense suppression of the cytochrome *bf* complex in the chloroplast electron transport chain confirmed the link between CO_2_ assimilation rate and the potential electron transport rate, *J* (Price et al. 1998; Yamori et al. 2011). Particularly useful were transgenic tobacco plants with reduced amounts of Rubisco where measurements of *A/C*_*i*_ curves showed photosynthesis to be limited solely by Rubisco even at high *C*_i_. These plants were used to generate in vivo values of Rubisco kinetic parameters from *A/C*_*i*_ curves measured under different O_2_ partial pressures and temperatures (von Caemmerer et al. 1994; Bernacchi et al. 2003). This allowed us to update values of the Rubisco kinetic constants in the C_3_ photosynthesis model by Farquhar et al. (1980) which had previously been sourced from measurements made on different species. With the aim of engineering plants with better Rubisco to improve photosynthesis, *A/C*_*i*_ curves were once again useful in analysing transgenic plants generated with foreign Rubiscos (Whitney et al. 1999; Whitney and Andrews 2003; Sharwood et al. 2008).

### The importance of mesophyll conductance

Von Caemmerer and Farquhar (1981) developed equations that calculated the intercellular [CO_2_] in the substomatal cavity and assumed that the difference between *C*_*i*_ and the CO_2_ partial pressure in the chloroplast was small such that it could be ignored. However, during CO_2_ assimilation, a [CO_2_] gradient must exist from the substomatal cavities across the cell wall, plasma and chloroplast membranes. This led to the definition of the term mesophyll conductance, *g*_*m*_*,* which quantifies the ease with which CO_2_ diffuses from intercellular airspace within a leaf to the sites of Rubisco carboxylation within chloroplasts. There was renewed interest in quantifying this mesophyll conductance, *g*_*m*_, when Evans et al. (1986) demonstrated that short-term measurements of carbon isotope discrimination concurrent with gas exchange measurement enabled estimation of *g*_*m*_. These initial measurements were made on a modified version of the gas exchange system shown in Fig. 1. Understanding the biology of mesophyll conductance is currently an active research field (Evans 2021). The wide range of different temperature responses across species makes it a challenge to incorporate mesophyll conductance into earth system models (von Caemmerer and Evans 2015; Rogers et al. 2017). Interestingly, progress has been made in genetically increasing mesophyll conductance for crop improvement (Ermakova et al. 2021; Salesse-Smith et al. 2024).

## Data Availability

N/A.
